# PGC-1α overexpression suppresses blood pressure elevation in DOCA-salt hypertensive mice

**DOI:** 10.1042/BSR20150076

**Published:** 2015-06-22

**Authors:** Qingbin Zhao, Junfang Zhang, Huifang Wang

**Affiliations:** * Department of Geratology, the First Affiliated Hospital of Xi’ an Jiaotong University, Xi’an, 710061 Shannxi, China; † Department of Internal Medicine, Chencang Hospital, Baoji, 721300 Shannxi, China

**Keywords:** deoxycorticosterone acetate (DOCA)-salt, endothelial dysfunction, endothelial nitric oxide synthase (eNOS), hypertension, peroxisome proliferator-activated receptor γ coactivator-1α (PGC-1α)

## Abstract

Increasing evidences have accumulated that endothelial dysfunction is involved in the pathogenesis of hypertension. Peroxisome proliferator-activated receptor γ (PPARγ) coactivator-1α (PGC-1α) has been identified as an essential factor that protects against endothelial dysfunction in vascular pathologies. However, the functional role of PGC-1α in hypertension is not well understood. Using an adenovirus infection model, we tested the hypothesis that PGC-1α overexpression retards the progression of hypertension in deoxycorticosterone acetate (DOCA)-salt mice model through preservation of the function of endothelium. We first demonstrated that PGC-1α expression not only in conductance and resistance arteries but also in endothelial cells was decreased after DOCA-salt treatment. In PGC-1α adenovirus-infected mice, the elevation of blood pressure in DOCA-salt mice was attenuated, as determined using tail-cuff measurement. Furthermore, PGC-1α overexpression inhibited the decrease in nitric oxide (NO) generation and the increase in superoxide anion (O_2_^−^) production in DOCA-salt-treated mice, in parallel with improved endothelium-dependent relaxation. Rather than affecting endothelial NO synthase (eNOS) total expression and phosphorylation, PGC-1α significantly inhibited eNOS uncoupling, as evidenced by increased eNOS homodimerization, BH4 levels, GTP-cyclohydrolase 1 (GTPCH1) and dihydrofolate reductase (DHFR) expression and heat-shock protein (Hsp)90–eNOS interaction. Our findings demonstrate that PGC-1α overexpression preserves eNOS coupling, enhances NO generation, improves endothelium-dependent relaxation and thus lowers blood pressure, suggesting that up-regulation of PGC-1α may be a novel strategy to prevent and treat hypertension.

## Abstract (precis)

PGC-1α over expression restores eNOS uncoupling by increasing BH4 levels and Hsp90/eNOS interaction, enhances NO generation, improves endothelium-dependently relaxation and thus lowers blood pressure. Our findings suggest that forced PGC-1α expression may be a novel strategy to prevent and treat hypertension.

## INTRODUCTION

Arterial hypertension is one of the major risk factors of cardiovascular diseases including stroke, coronary artery disease and heart failure and is a leading cause of morbidity and mortality globally [1,2]. Hypertension is directly associated with physiological changes in the vessel wall, characterized by fluid shear stress, turbulent blood flow, vascular smooth muscle cells (VSMCs) hyperplasia and endothelial dysfunction [3]. Endothelial dysfunction has been implicated in the pathogenesis and progression of hypertension, which results from an imbalance between endothelium-derived nitric oxide (NO) and reactive oxygen species (ROS) [4]. This imbalance finally leads to abnormal circulatory peripheral resistance and increase blood pressure [5].

Endothelial NO synthase (eNOS) is a key enzyme for the maintenance of vascular homoeostasis by producing NO. Among the several mechanisms involved in the regulations of eNOS activity, eNOS uncoupling plays a crucial role triggering the dysfunction of eNOS. Under certain pathological circumstances, eNOS can be uncoupled, which is mainly due to a reduced concentration of its cofactor, tetrahydrobiopterin (BH4) [5]. Additional researches provided evidences that Hsp90 is an essential regulator of eNOS uncoupling, because inhibition of heat-shock protein (Hsp)90 expression or Hsp90–eNOS interaction results in eNOS uncoupling with a decrease in enzymatic activity and NO generation [6–8]. Interestingly, previous studies have reported that uncoupled eNOS is a typical feature in deoxycorticosterone acetate (DOCA)-salt mice [9,10], suggesting eNOS uncoupling plays an important role in salt-sensitive low-renin hypertension. Therefore, an understanding of the dynamic regulation of eNOS coupling is essential to elucidate the contribution of endothelial dysfunction in hypertension.

Peroxisome proliferator-activated receptor γ (PPARγ) coactivator-1α (PGC-1α) is the first member to be identified in PGC-1α family [11], which has been suggested to be a master regulator of mitochondrial biogenesis and energy metabolism [12,13]. Recent studies have demonstrated that PGC-1α plays a crucial role in the development of cardiovascular diseases. For example, PGC-1α has been found to be an essential regulator of oxidative stress in cardiac cells [14,15], as evidenced by the development of cardiac dysfunction in PGC-1α knockout mice [16]. On the other hand, current research revealed anti-atherosclerotic effects of PGC-1α [17,18], which was due at least in part to an improvement in endothelial dysfunction [19]. However, the functional role of PGC-1α in the progression of hypertension remains unknown.

Interestingly, in the present study, we found PGC-1α was decreased in endothelium of aortas isolated form DOCA-salt hypertensive mice. Therefore, we aim to investigate the direct link between of PGC-1α and hypertension and the underlying molecular mechanisms. Our study provides evidences that the inhibitory effect on eNOS uncoupling underlies, at least partially, the anti-hypertensive effects of PGC-1α.

## MATERIALS AND METHODS

### Materials and reagents

Dulbecco's modified Eagle's medium (DMEM)/F-12 medium, FBS and dihydroethidium (DHE) were purchased from Invitrogen. Mouse PGC-1α adenovirus (Ad-PGC-1α) and Lacz were constructed and purchased from Sunbio Medical Biotechnology. Protein A/G agarose, primary antibodies targeting PGC-1α, eNOS, Hsp90, glyceraldehyde-3-phosphate dehydrogenase (GAPDH), GTP-cyclohydrolase 1 (GTPCH1) and dihydrofolate reductase (DHFR) were from Santa Cruz Biotechnology. Phosphorylated eNOS (Ser^1177^) antibody was obtained from Cell Signaling Technology. All other reagents utilized were from Sigma Chemical Co. unless otherwise specified.

### DOCA-salt hypertensive model

All animal procedures were approved by the Committee on the Ethics of Animal Experiments of Xi’ an Jiaotong University and in accordance with the ‘Guide for the Care and Use of Laboratory Animals’ of the National Institute of Health in China.

Twelve-week-old (20–25 g) male C57BL/6 mice were obtained from Jackson Laboratory. DOCA-salt hypertension model was established as previously [5]. Briefly, mice received uninephrectomy under anaesthesia with isoflurane. A slow-release DOCA pellet (50 mg, Innovative Research of America) was implanted subcutaneously through a 1-cm incision between the shoulder blades. DOCA-salt animals were given 1% NaCl and 0.2% KCl drinking water at the beginning of DOCA treatment. Control animals were sham-operated and were given normal drinking and received no DOCA pellet. After 7 days of DOCA treatment, sham and DOCA-salt mice were randomized to injected with Lacz (10^9^ pfu/mouse) or Ad-PGC-1α (10^9^ pfu/mouse) via tail vein once daily for the following 14 days. Four groups of animals were examined: Lacz + Sham, Ad-PGC-1α + Sham, Lacz + DOCA and Ad-PGC-1α + DOCA. Systolic blood pressure (SBP) was measured in conscious mice by tail-cuff plethysmography (BP-98A, Softron Co) during a fixed time period of the day (8:00–10:00 a.m.).

### Cell culture

To isolate mouse aortic endothelial cells (MAECs), aorta was harvested from mouse under isoflurane-anaesthesia and immersed immediately in the Kreb's solution (137 mmol/l NaCl, 5.4 mmol/l KCl, 2.0 mmol/l CaCl_2_, 1.1 mmol/l MgCl_2_, 0.4 mmol/l NaH_2_PO_4_, 5.6 mmol/l glucose, 11.9 mmol/l NaHCO_3,_ pH=7.2). The aorta was opened longitudinally and cut into 3-mm long sections and plated on a matrigel pre-coated dish. DMEM/F-12 medium with supplemented growth factors was added to the dish. After 7–10 days, cells began to outgrow and then were sub-cultured when reaching confluence.

### Western blot analysis

Homogenates of mouse aortas and mesenteric resistance arteries and cell extracts were subjected to western blot analysis as described previously [5,20]. Briefly, equal amount of protein was size-fractionated electrophoretically using SDS/PAGE and transferred on to nitrocellulose membrane (Millipore). The membranes were incubated with targeted primary antibodies and blotted with respective secondary antibodies. Bands were visualized by an ECL kit (Beyotime) and quantified by ImageJ software (NIH). To detect eNOS monomer–dimer formation, low temperature western blot assay was performed. Aortas were homogenized and protein samples without thermal denaturation were subjected to SDS/PAGE (6% gel) at 4°C.

### RNA isolation and analysis

Total RNA from mouse aortas or mesenteric resistance arteries or MAECs was isolated using RNeasy Micro Kit (Qiagen) according to the manufacturer's instructions. Reverse transcription was performed using the ReverTra ACE qPCR RT Kit (Toyobo) and PCR amplification was carried out with the ABI Prism 7000 sequence detection (Applied Biosystem). Sequence-specific primers and SYBR Green PCR master mix were obtained from Invitrogen. The primer sequences used to amplify mouse PGC-1α cDNA were 5′-TCACTAACGTGTGGGTCAGC-3′ (sense) and 5′-GCTCTCATGTTGCCAAAGCC-3′ (antisense); primers for GAPDH were 5′-CTTCACCACCATGGAGAAGGC-3′ (sense) and 5′-GGCATGGACTGTGGTCATGAG-3′ (antisense). Amplification was carried out by 32 cycles of 95°C for 30 s and 59°C for 1 min after an initial 5 min at 95°C.

### Reactivity experiments

*In vitro* arterial preparation was used to study the reactivity of mouse aorta and first-order branches of the mesenteric resistance artery as described previously [21]. Briefly, mice were anaesthetized and the artery was dissected and placed immediately in the ice-cold Kreb's solution. After the surrounding fat tissue was carefully cleaned, arterial segments (2–3 mm long) were prepared and mounted as a ring in warm Kreb's solution gassed with 95% O_2_ and 5% CO_2_ at 37°C continuously. All rings were stretched to a resting tension of 5 mN in bath medium and allowed a 1 h equilibration period to reach a stable resting diameter. After preparation, endothelium-dependent relaxation was determined with cumulative doses of acetylcholine (Ach, 10^−9^–3 × 10^−5^ mol/l) after precontraction with phenylephrine (Phe, 10^−5^ mol/L). NO-mediated relaxation was verified by measuring the response to Ach after pre-incubation with the eNOS inhibitor *N*(G)-nitro-L-arginine (L-NNA, 10^−4^ mol/l). Endothelium-independent relaxation was assayed by measuring the relaxation response to sodium nitroprusside (SNP; 10^−10^–10^−6^ mol/l). Concentration-response curves for phenylalanine were conducted over the range of 10^−9^–10^−5^ mol/l and expressed as percentage of the maximal contraction induced by KCl (5 × 10^−4^ mol/l).

### Measurement of cGMP and nitrite production

MAECs were incubated with 0.75 ml of Kreb's solution containing A23187 (5 μM) for 30 min and washed with cold PBS three times and then lysed in radioimmunoprecipitation assay (RIPA) buffer (Beyotime) containing protease and phosphatase inhibitor cocktail (Merck). The concentrations of cGMP and nitrite in cell lysates were determined using cGMP ELISA Kit (R&D Systems) and total NO assay kit (Beyotime) respectively. All measurements were performed as recommend by the manufacturer's protocol.

### Superoxide anion production measurements

Detection of O_2_^−^ in thoracic aorta and MAECs was performed using DHE fluorescent dye. MAECs were seeded in six-well plates or 96-well bottom black plates. To verify the role of mitochondria and NADPH oxidase and eNOS in O_2_^−^ production, MAECs were pre-incubated with PEG–superoxide dismutase (PEG–SOD;10^−4^ mol/l) or apocynin (10^−6^ mol/l) or L-NNA (10^−6^ mol/l) for 30 min before detection respectively. After treatment, frozen thoracic aorta slides (5 μm) and MAECs were stained by DHE (5 μmol/l) in PBS buffer and incubated in a light-protected chamber at 37°C for 30 min. Images were obtained using a fluorescent microscope (Zeiss) at 488 nm excitation and 525 nm emission wavelengths. For quantification of O_2_^−^ level in tissue, fluorescence (intensity × area) was measured using NIH ImageJ software (in arbitrary units). For quantification of O_2_^−^ level in MAECs, the fluorescence intensity of the 96-well plates was analysed using a Microplate Reader (Infinite F500).

### Detection of mitochondrial reactive oxygen species generation

MitoSOX red reagent was used to measure the mitochondrial ROS (mROS) generation. MAECs were incubated with MitoSOX Red (5 μmol/l) in serum-free DMEM/F-12 medium in the dark at 37°C for 30 min. After incubation, the cells were gently washed with warm PBS and visualized under a fluorescent microscope at 370 nm excitation and 420 nm emission wavelengths.

### Detection of NADPH oxidase activity

MAECs were washed twice with PBS and scraped into NADPH lysis buffer (1.0 mol/l K_2_HPO_4_, 0.1 mol/l EGTA, 0.15 mol/l and protease inhibitor cocktail, pH 7.0) Cell lyates were sonicated with three cycles of 6-s bursts on ice and centrifuged at 12000 ***g*** for 5 min. Then the supernatant was incubated with lucigenin (5 mmol/l) for 10 min at 37°C in dark. The basal relative light units (RLU) of chemiluminescence were determined by a luminometer (Promega) every 10 s for 1 min. NADPH (100 μmol/l) was immediately added to the suspension to start the reaction and the chemiluminescence was determined for another 1 min as experimental RLU. The NADPH oxidase activity was expressed as RLU/s·mg protein.

### Measurement of BH4 levels

Arterial BH4 levels were determined by HPLC with fluorescence detection as previously described [5]. Briefly, 10 μl of a mixture (1.5 mol/l HClO_4_:2 mol/l H_3_PO_4_, 1:1) was added to 90 μl of aortas homogenates to remove proteins. For total biopterins (BH4, BH2 and oxidized biopterins) determination, 10 μl of 1% iodine in 2% KI solution was added to 90 μl of protein-free supernatant. For BH2 and biopterins determination, 10 μl of 1 mol/l NaOH was added to 80 μl of homogenates, followed by adding 10 μl of 1% iodine in 2% KI solution. After centrifugation, 10 μl of supernatant was injected into a 150-mm long, 4.6-mm-inner diameter welchrom-C18 column with a reverse phase HPLC system (Shimadzu, Class-VP). Fluorescence detection (350 nm excitation, 450 nm emission) was performed using a RF10AXL fluorescence detector (Shimadzu). BH4 levels, expressed as pmol/mg protein, were calculated by subtracting BH2 plus biopterins from total biopterins.

### Immunoprecipitation

Thoracic aortas were homogenized in lysis buffer and centrifuged at 12000 ***g*** for 15 min. The lysates were pre-cleared non-specific binding by incubation with protein A/G agarose beads for 1 h. After centrifugation, the pre-cleared supernatants were then co-incubated with eNOS antibody and protein A/G agarose beads at 4°C overnight. The immunoprecipitates were washed extensively with PBS three times and subjected to SDS/PAGE using Hsp90 antibody as previously described.

### Statistical analysis

All data were given as value±S.E.M. Data were analysed using the one-way ANOVA or an unpaired Student's *t*test by SPSS 17.0 statistical software (SPSS Inc.). *P*-values less than 0.05 were considered to be statistically significant.

## RESULTS

### PGC-1**α** expression is reduced in DOCA-salt hypertension

In aortas isolated from DOCA-salt-treated mice, the protein and mRNA levels of PGC-1α were both significantly decreased compared with sham-operated mice (Figures 1A and 1B). Similar tendency was observed in mesenteric resistance arteries isolated from sham or DOCA-salt mice (Supplementary Figures S1A and S1B). Intriguingly, western blot showed that PGC-1α expression in arteries which were stripped from endothelium was indistinguishable between sham-operated and DOCA-salt-treated mice (Supplementary Figures S2A and S2B), suggesting DOCA-salt-decreased PGC-1α expression may be an endothelial-specific event. To further confirm the down-regulation of PGC-1α in endothelium induced by DOCA-salt treatment, we analysed the PGC-1α expression in MAECs isolated from sham-operated and DOCA-salt-treated mice. Consistent with the previously data, the protein and mRNA levels of PGC-1α in MAECs were decreased after DOCA-salt treatment (Supplementary Figures S2C and S2D), indicating that PGC-1α expression in endothelium may be negatively correlated with the development of hypertension induced by DOCA-salt.

**Figure 1:**
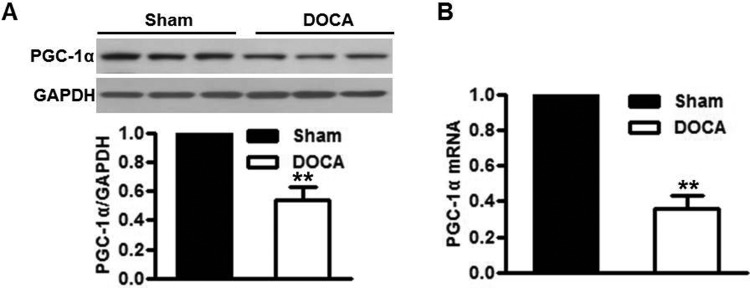
Reduced PGC-1**α** is found in the aortas of DOCA-salt hypertensive mice (**A** and **B**) PGC-1α expression in aortas of mice treated with DOCA-salt over a 3-week period was determined by western blot (**A**) and quantitative PCR (**B**). **P<0.01 compared with sham group, *n*=10/group.

### PGC-1**α** restrains the elevation of blood pressure in DOCA-salt hypertension model

Base on the decrease in PGC-1α after DOCA-salt treatment, we speculated that PGC-1α may be involved in the progression of hypertension. To verify this assumption, we *in vivo* established a DOCA-salt hypertension model with a gene approach. C57BL/6 mice were injected with Ad-PGC-1α or an adenovirus vector Lacz with or without DOCA-salt treatment (Supplementary Figure S3A). The infection efficiency of Ad-PGC-1α was confirmed in aortas and MAECs by western blot (Supplementary Figure S3B). As shown in Figure 2(A), no significant differences were noticed in baseline SBP among the groups. Moreover, infection with Ad-PGC-1α produced no obvious effects on SBP in sham mice over a 3-week period. Compared with sham-operated mice, SBP was increased dramatically in Lacz-infected DOCA-salt-treated mice during the time course. However, the elevation of SBP was inhibited in Ad-PGC-1α-infected DOCA-salt-treated mice. The significant differences between these groups were observed starting on day 12 and continuing through day 21. Body weight was similar between Lacz-infected and Ad-PGC-1α-infected mice before DOCA-salt treatment. Three-week DOCA-salt treatment showed a significant reduction in body weight in Lacz-infected mice, which was remarkably alleviated in DOCA-salt Ad-PGC-1α-infected mice (Figure 2B).

**Figure 2:**
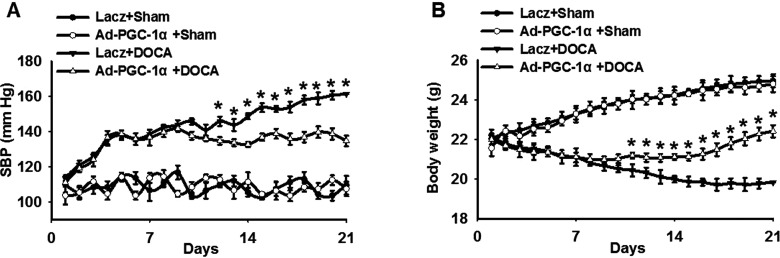
PGC-1**α** overexpression attenuates blood pressure elevation in DOCA-salt hypertensive mice (**A**) Average SBP in Lacz-infected and Ad-PGC-1α-infected mice with or without DOCA-salt treatment, measured by non-invasive tail-cuff methods. (**B**) The weights of mice among the groups. *P<0.05, Lacz + DOCA compared with Ad-PGC-1α + DOCA, *n*=6/group.

### PGC-1**α** ameliorates the decreased NO generation and the impaired vasorelaxation in DOCA-salt hypertension

We investigated whether the inhibition of blood pressure following overexpression of PGC-1α was associated with an increase in NO generation. cGMP and nitrite concentration in MAECs were measured with or without A23187 treatment. Under basal level, NO generation before DOCA-salt treatment was indistinguishable between Lacz-infected and Ad-PGC-1α-infected mice. DOCA-salt treatment presented a sharp decrease in cGMP and nitrite concentration, which was almost completely abolished in DOCA-salt Ad-PGC-1α-infected mice. A23187, a calcium ionophore, increased NO generation in sham-operated mice, but no significant differences were observed between Lacz-infected and Ad-PGC-1α-infected groups. In DOCA-salt Lacz-infected mice, A23187 increased NO generation to a significantly lesser extent than in DOCA-salt Ad-PGC-1α-infected mice (Figures 3A and 3B), suggesting that PGC-1α is important for NO bioavailability during Ca^2+^-induced activation. To further explore the functional impact of PGC-1α-preserved NO generation, we studied endothelium-dependent relaxation to acetylcholine (Ach, 10^−9^–3 × 10^−5^ mol/l) in thoracic aortas and mesenteric resistance arteries. In sham mice, the concentration-response curves to Ach were similar between Lacz and Ad-PGC-1α group. However, the endothelium-dependent relaxation was significantly impaired in Lacz-infected mice after DOCA-salt treatment. In contrast, this impairment was obviously improved in DOCA-salt Ad-PGC-1α-infected mice (Figures 3C and 3D). The relaxation response to Ach was almost completely eliminated by L-NNA (10^−4^ mol/l; Supplementary Figures S4A and S4C). Moreover, endothelium-independent relaxation in response to SNP (10^−10^–10^−6^ mol/l) revealed no significantly differences among the groups (Supplementary Figures S4B and S4D). The phenylephrine (Phe, 10^−9^–10^−5^ mol/l)-induced contractile responses seemed similar and no difference was observed in thoracic aortas (Figure 3E). Intriguingly, in mesenteric resistance arteries, DOCA-salt treatment increased contractile responses to phenylephrine in Lacz-infected mice, but not in Ad-PGC-1α-infected mice. Accordingly, this difference was eliminated after pretreatment with L-NNA (Figure 3F), suggesting that the lack of effect of DOCA-salt on phenylephrine response in resistance artery of Ad-PGC-1α-infected mice may be ascribed to a lesser decrease in NO generation. Collectively, these observations indicate that overexpression of PGC-1α improves endothelium-dependent relaxation.

**Figure 3:**
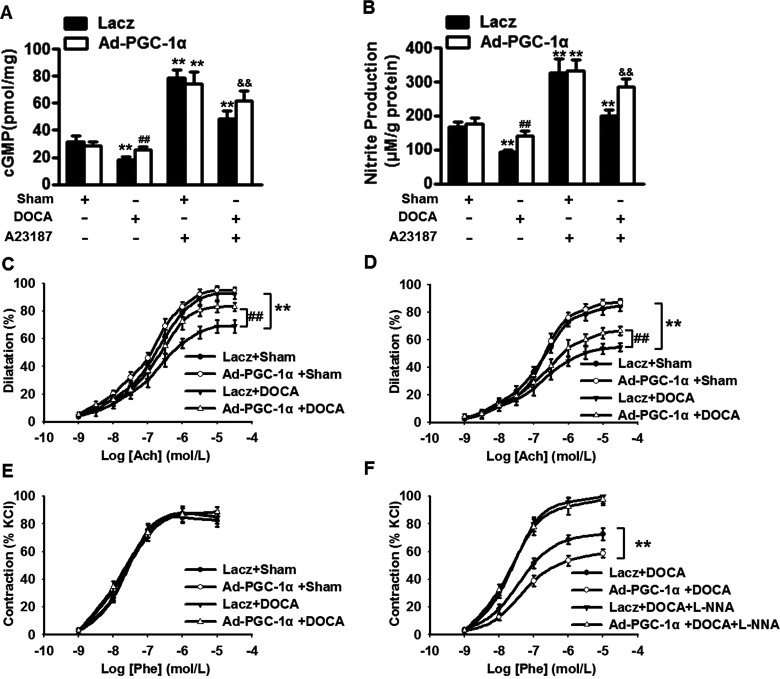
PGC-1**α** ameliorates the impairment of NO generation and endothelium-dependent relaxation in DOCA-salt hypertensive mice (**A** and **B**) After incubation with A23187 (5 μM) for 30 min, NO generation in MAECs were determined by measuring cGMP (**A**) and nitrite production (**B**). *n*=6. **P<0.01 compared with Lacz + sham; ^##^*P*<0.01 compared with Lacz + DOCA; ^&&^*P*<0.01 compared with Lacz + DOCA + A23187. (**C** and **D**) Endothelium-dependent relaxation to acetylcholine (Ach, 10^−9^–10^−5^ mol/l) in thoracic aortas (**C**) and mesenteric resistance arteries (**D**). (**E** and **F**) Concentration–response curves to Phe (10^−5^ mol/l) in thoracic aortas (**E**) and mesenteric resistance arteries (**F**). *n*=8/group. **P<0.01 compared with Lacz + sham; ^##^*P*<0.01 compared with Lacz + DOCA.

### PGC-1**α** inhibits O_2_^−^ production in DOCA-salt hypertension

O_2_^−^ production was determined by assay DHE imaging. Figure 4 (A) showed sections from mice thoracic aortas, taken from Lacz-infected or Ad-PGC-1α-infected mice before and after DOCA-salt treatment. DHE staining revealed that infection with Ad-PGC-1α produced no effects on O_2_^−^ production in sham mice. Compare with sham mice, the fluorescence intensity in Lacz-infected DOCA-salt-treated mice was elevated to nearly 4-fold; this was dramatically inhibited after infection with Ad-PGC-1α. PEG-SOD, which was used as a positive control, significantly attenuated DOCA-salt-induced O_2_^−^ production (Figure 4B). Similar results were observed in MAECs isolated from there four groups (Figures 4C and 4D). Of note, the increased O_2_^−^ production in MAECS of Lacz-infected DOCA-salt-treated mice was decreased significantly by PEG–SOD or apocynin or L-NNA, suggesting mitochondria, NADPH oxidase and eNOS uncoupling may be the main source of O_2_^−^.

**Figure 4:**
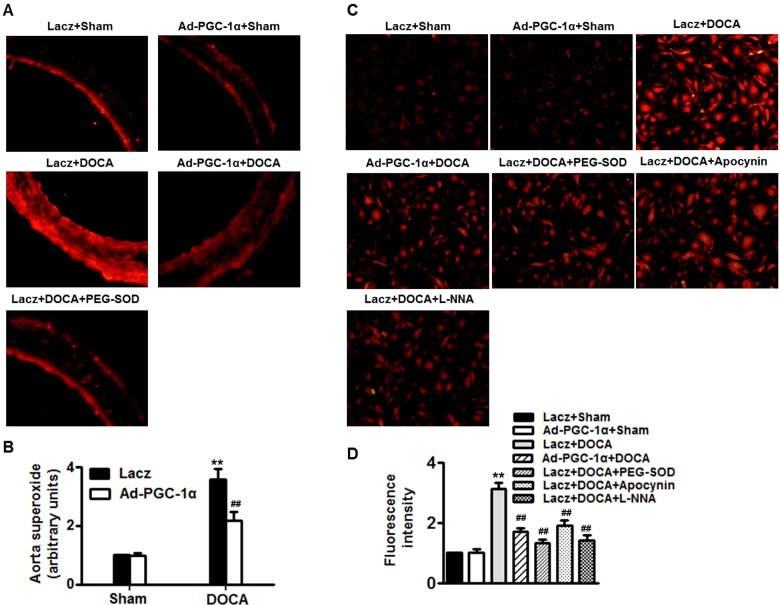
PGC-1**α** decreases O_2_^−^ levels in DOCA-salt hypertension (**A** and **B**) O_2_^−^ in aorta sections was determined by DHE fluorescence (×400; **A**) and quantitative evaluation of DHE fluorescence intensity was performed (**B**). (**C** and **D**) MAECs were isolated from four groups of mice as mentioned in methods section. Additionally, MAECs isolated from DOCA-salt mice were pre-incubated with PEG–SOD (10^−4^ mol/l) or apocynin (10^−6^ mol/l) or L-NNA (10^−6^ mol/l) for 30 min before O_2_^−^ detection, respectively (×200). (**D**) Quantitative evaluation of DHE fluorescence intensity in MAECs was performed. *n*=5–7/group. **P<0.01 compared with Lacz + sham; ^##^*P*<0.01 compared with Lacz + DOCA.

### PGC-1**α** prevents eNOS uncoupling in DOCA-salt hypertensive mice

Although DOCA-salt significantly induced mROS generation and NADPH oxidase activity, these elevations did not change after overexpression of PGC-1α (Supplementary Figure S5). The data excluded the possibility that mitochondrial sources and NADPH oxidase pathway involved in the protective effects of PGC-1α overexpression. Next, we investigated whether PGC-1α is involved in the regulation of NO generation due to eNOS expression and activity, eNOS expression, phosphorylation and dimerization were measured (Figure 5A). Western blotting showed that total eNOS expression in aortas remained unchanged among groups (Figure 5B). Although the ratio of phosphorylated eNOS to total eNOS at the Ser^1177^ residue was decreased in Lacz-infected DOCA-salt treatment, Ad-PGC-1α infection did not reverse the decreased eNOS phosphorylation (Figure 5C). Notably, when the active (dimer) and inactive forms (monomer) of eNOS were determined, it was found that the eNOS dimer-monomer ratio in Lacz-infected DOCA-salt-treated mice was reduced by over 50%. When the mice were infected with Ad-PGC-1α, the ratio of eNOS dimer to monomer was almost recovered (Figure 5D). As shown in Figure 5(E), DOCA-salt treatment resulted in a significant decrease in BH4 concentration; this decrease was remarkably inhibited after infection with Ad-PGC-1α. These resulted were further confirmed by measuring the expressions of GTPCH1 and DHFR, both of which are involved in BH4 synthesis (Supplementary Figures S6A and S6B). Additionally, we immunoprecipitated eNOS from aortas homogenates and compared the Hsp90/immunoprecipitated eNOS ratio among the groups. DOCA-salt reduced Hsp90–eNOS interaction in Lacz-infected mice by more than 50%. The interaction was much stronger in Ad-PGC-1α-infected mice (Figure 5F). Together, these data indicate that PGC-1 prevents eNOS uncoupling by augmentation of BH4 concentration and Hsp90–eNOS interaction.

**Figure 5:**
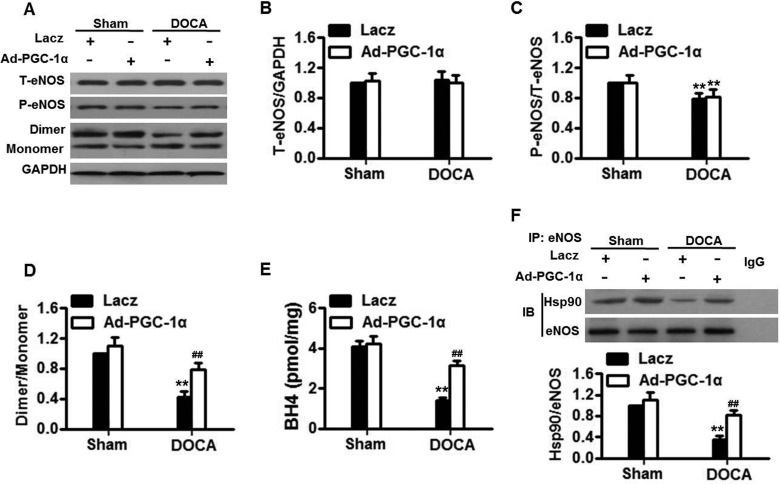
PGC-1**α** inhibits eNOS uncoupling in DOCA-salt hypertensive mice (**A**) Representative western blots of total eNOS (T-eNOS) expression, phosphorylated eNOS at the Ser^1177^ (P-eNOS) and eNOS dimerization in aortas isolated from Lacz-infected and Ad-PGC-1α-infected mice with or without DOCA-salt treatment. (**B**–**D**) Densitometric analysis of eNOS expression (**B**), phosphorylation (**C**) and dimerization (**D**). (**E**) BH4 concentration in aortas homogenates was measured. (**D**) Immunoblotting for Hsp90 or eNOS after immunoprecipitation (IP) with eNOS antibody in aortas homogenates. *n*=5–7/group. **P<0.01 compared with Lacz + sham; ^##^*P*<0.01 compared with Lacz + DOCA.

## DISCUSSION

To our knowledge, the present study demonstrates for the first time that (1) PGC-1α expression was decreased after DOCA-salt treatment. (2) The progression of hypertension was retarded in Ad-PGC-1α-infected DOCA-salt-treated mice, which may be related to an improvement in endothelium-dependent relaxation by increasing NO generation and reducing O_2_^−^ production. (3) Overexpression of PGC-1α inhibited eNOS uncoupling but not eNOS dephosphorylation of Ser^1177^, as demonstrated by restored the decrease in BH4 levels and Hsp90–eNOS interaction.

PGC-1α is an important regulator involved not only in cellular and systemic metabolism but also in vascular biology [17,22]. Recent growing evidences have demonstrated the relevance of PGC-1α expression in several pathological processes of the cardiovascular system, including VSMCs hyperplasia, inflammation, foam cell formation and endothelial dysfunction [17,19,23,24]. In the present study, we found that PGC-1α expression was decreased in DOCA-salt hypertensive mice in both conductance and resistance arteries, suggesting that down-regulation of PGC-1α may be correlated with the development of hypertension. Indeed, DOCA-salt-induced the elevation of blood pressure was significantly inhibited in Ad-PGC-1α-infected mice compared with Lacz-infected mice. Of note, it was demonstrated that PGC-1α expression was positive associated with the maximal response to Ach [18], suggesting PGC-1α may play a crucial role in regulating NO generation and vascular relaxation and thus the development of hypertension. In the present study, our results showed that overexpression of PGC-1α restored DOCA-salt-induced the decrease in NO generation, as demonstrated not only by cGMP concentration but also by nitrite production. We further observed that PGC-1α improved endothelium-dependent relaxation in both conductance and resistance arteries. Interestingly, overexpression of PGC-1α produced different effects on contractile responses in the conductance and resistance vasculature, indicating that specific response of different vasculature should be noted. It is not surprised since L-NNA incubation can ablate the decrease in phenylephrine contraction in mesenteric resistance arteries isolated from Ad- PGC-1α-infected mice, further demonstrating that endothelium-derived NO generation, an important determinant of blood pressure, after DOCA salt treatment was better preserved in resistance vasculature of Ad- PGC-1α-infected mice.

Despite increasing evidence supporting that the activity of eNOS is regulated by multiple mechanisms that include transcriptional regulation by its promoter, post-transcriptional regulation of mRNA stability and post-translational regulation by coupling and phosphorylation [21], the mechanisms by which eNOS dysfunction initiates hypertension are not well understand. Our results, together with previous evidence demonstrated that total expression of eNOS was similar, but eNOS phosphorylation (Ser^1177^) was decreased [5]. Interestingly, despite the enhanced NO generation, overexpression of PGC-1α did not inhibit the dephosphorylation of eNOS, suggesting eNOS expression and phosphorylation are not involved in this process. Notably, it has been well-documented that DOCA-salt may act by regulating the dimerization of eNOS rather than increase total eNOS enzymes [9,10]. Failure of eNOS to couple oxygen to L-arginine metabolism resulted in eNOS-derived O_2_^−^ rather than NO, which is a cardinal feature of endothelial dysfunction [25]. Therefore, eNOS uncoupling contributes to endothelial dysfunction by an imbalance between NO generation and O_2_^−^ production. In the present study, our data showed that arterial O_2_^−^ was significantly increased in Lac-infected DOCA-salt-treated mice compared with Lacz-infected sham mice and this was obviously diminished after infection with Ad-PGC-1α. Similar results were obtained in MAECs isolated from mice among the groups and we also found the increased O_2_^−^ production induced by DOCA-salt was inhibited by eNOS inhibitor, L-NNA. Our results were in agreement with previous studies in aortas [5,9] and further confirmed uncoupled eNOS is a major source of O_2_^−^ production observed in DOCA-salt hypertension model. Thus, it is reasonable to assume that PGC-1α could have a dramatic effect on eNOS dimerization. We observed that the ratio of dimer to monomer was decreased after DOCA-salt treatment. However, the decreased ratio was normal in Ad-PGC-1α-infected mice. These data suggest that the inhibition of eNOS uncoupling underlies, at least in part, the anti-hypertension effect of PGC-1α in salt-sensitive low-renin hypertension.

eNOS uncoupling has been reported as a consequence of reduced BH4 concentration or Hsp90–eNOS complex formation [5,8]. BH4 plays a determining role in the regulation of eNOS-modulated endothelial response and inadequate supply of cellular BH4 is directly associated with eNOS uncoupling [26]. In the present study, we evidenced that PGC-1α inhibited DOCA-salt-induced the decease of BH4 levels. This is supported by the finding that overexpression of PGC-1α counteracted the inhibitory effect of DOCA-salt on expression of GTPCH1 and DHFR, indicating PGC-1α maintain adequate intracellular BH4 concentration by increasing the expression of GTPCH1 and DHFR. In addition to BH4, Hsp90 has a regulatory effect on eNOS function, in which its association with eNOS favours NO generation [27]. We found PGC-1α restored the decrease in Hsp90–eNOS interaction after DOCA-salt challenge. The stimulatory effect of PGC-1α on BH4 levels and Hsp90–eNOS interaction has not been reported previously.

In summary, we demonstrate that PGC-1α overexpression restores eNOS uncoupling probably by increasing BH4 levels and Hsp90–eNOS interaction, in turn, enhances NO generation and improves endothelium-dependently relaxation and thus lowers blood pressure. Our work reveals a novel role of PGC-1α in the development of hypertension, suggesting that forced PGC-1α expression may be a novel approach for the treatment of hypertension.

## Abbreviations

Ad-PGC-1α: PGC-1α adenovirus
BH4: tetrahydrobiopterin
DHE: dihydroethidium
DHFR: dihydrofolate reductase
DMEM: Dulbecco's modified Eagle's medium
DOCA: deoxycorticosterone acetate
eNOS: endothelial NO synthase
GAPDH: glyceraldehyde-3-phosphate dehydrogenase
GTPCH1: GTP-cyclohydrolase 1
Hsp: heat-shock protein
L-NNA: *N*(G)-nitro-L-arginine
MAEC: mouse aortic endothelial cell
mROS: mitochondrial ROS
NO: nitric oxide
PGC-1α: PPARγ coactivator-1α
Phe: phenylephrine
PPARγ: peroxisome proliferator-activated receptor γ
RLU: relative light units
ROS: reactive oxygen species
SBP: systolic blood pressure
SNP: sodium nitroprusside
SOD: superoxide dismutase
VSMC: vascular smooth muscle cell
